# Evaluating the Anti‐Oxidant and Anti‐Inflammatory Properties of Watercress Supplementation at Short‐Term Follow‐Up: A Systematic Review of Randomized Controlled Trials

**DOI:** 10.1002/fsn3.70407

**Published:** 2025-06-05

**Authors:** Jimmy Wen, Muhammad Karabala, Zohaer Muttalib, Burhaan Syed, Ramy Khalil, Daniel Razick, Adam Razick, David Pai

**Affiliations:** ^1^ California Northstate University College of Medicine Elk Grove California USA; ^2^ University of California Los Angeles Los Angeles California USA

**Keywords:** anti‐inflammatory, anti‐oxidant, *Nasturtium officinale*, systematic review, watercress

## Abstract

Watercress (WC) has been used extensively in traditional medicine for various healthcare conditions such as hypertension, hyperglycemia, arthritis, and more. This systematic review evaluates WC's antioxidant and anti‐inflammatory markers in human studies. A systematic review search was conducted in PubMed, Embase, and Cochrane Library for studies reporting anti‐inflammatory and anti‐oxidative markers following WC supplementation, following the Preferred Reporting Items for Systematic Reviews and Meta‐Analyses (PRISMA) guidelines. Study variables included the number of patients, dosage/formulation of WC, mean age, and follow‐up time, pre‐ and post‐intervention antioxidant/anti‐inflammatory outcomes, and complications. Seven RCTs, including 302 patients with a mean age of 47 years (23–61) and a mean follow‐up time of 39 days (21–60), were included in this study. The dosage varied from 85 g/day to 750 mg/kg/day. Antioxidant parameters reported included superoxide dismutase (five studies), ferric‐reducing antioxidant power (two studies), glutathione peroxidase (two studies), retinol (two studies), β‐carotene (two studies), and α‐tocopherol (two studies). Oxidative stress parameters included protein carbonyls (four studies), thiobarbituric acid reactive substances (two studies), malondialdehyde (two studies), nitric oxide (two studies), T‐SH (two studies), and CAT (three studies). Inflammatory markers included two studies reporting on tumor necrosis factor‐a (two studies) and one study reporting on interleukin‐1, interleukin‐6, and C‐reactive protein. WC administration demonstrated improvement for most antioxidant and anti‐inflammatory markers and had a strong safety profile. WC had a positive effect on anti‐oxidant and anti‐inflammatory markers. However, the relatively short follow‐up times and heterogeneous patient demographics and formulations/dosages of WC warrant further studies to assess the benefits of WC.

## Graphical abstract

This systematic review highlights the antioxidant and anti‐inflammatory effects of watercress (WC) supplementation in randomized controlled trials. Despite the heterogeneity in WC formulations, dosage, follow‐up, and outcomes, WC demonstrated improvements in biomarkers such as IL‐1, IL‐6, TNF‐α, and antioxidant enzymes. These effects are mediated through bioactive compounds in WC, such as glucosinolates, carotenoids, and flavonoids, which modulate molecular pathways including Nrf2, NF‐kb, and PI3k/Akt.

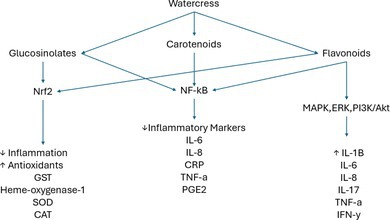

## 1 Introduction

Chronic low‐grade inflammation and oxidative stress are commonly observed in many common diseases such as diabetes, cardiovascular disease, obesity, metabolic syndrome, geriatric conditions, and cachexia (Peluso et al. 2013). Inflammatory markers are significant predictors of mortality in these conditions, particularly interleukin‐6 (IL‐6) and tumor necrosis factor‐alpha (TNF‐α) (Peluso et al. 2013). Thus, therapeutic research into the blockade of these inflammatory markers has been of great interest (Walsh et al. 2023). In addition, dietary intake has also been suggested to modulate levels of these markers, representing a potentially significant clinical implication. Plant‐based diets have decreased the risk of mortality from inflammatory‐mediated diseases and serum concentrations of inflammatory markers (Syed et al. 2023; Zhang et al. 2024). Epidemiological and human studies have shown that cruciferous vegetables can reduce oxidative stress and the risk of developing chronic diseases (Clemente et al. 2021).

Watercress (WC) is a cruciferous vegetable that is part of the Brassicaceae family and generally grows around water sources like rivers, ponds, and streams (Keser and Saygideger 2010). It is native to Western Asia, Europe, India, and Africa, but can be grown anywhere. WC is highly valued for its high vitamin (B1, B2, B3, B6, C, E), mineral (Calcium, Iron), polyphenol, isothiocyanate, terpene, and glucosinate levels (Clemente et al. 2021). WC has been used extensively in traditional medicine to target hypertension, hyperglycemia, arthritis, respiratory diseases, hypercholesterolemia, diuresis, and more (Panahi Kokhdan et al. 2021). Additionally, it has been used as an expectorant, odontalgic, and for anti‐estrogenic activity (Panahi Kokhdan et al. 2021).

Although the nutritional value of WC has been well researched, its biological properties in humans are currently still being investigated. In vitro, WC exhibits high anti‐oxidant capabilities and has been shown to inhibit the initiation, proliferation, and metastasis of cancer cell models (Giallourou et al. 2019). Many in vitro and pharmacological studies have demonstrated the anti‐oxidative and anti‐inflammatory effects of WC (Panahi Kokhdan et al. 2021). However, the literature has not comprehensively compiled WC's anti‐oxidant and anti‐inflammatory effects in human studies (Panahi Kokhdan et al. 2021). Thus, this systematic review aims to assess the anti‐oxidant and anti‐inflammatory properties of WC and to better understand the reduction of various inflammatory molecules through randomized controlled trials (RCTs) with human participants. We hypothesize that WC in humans, due to its various chemical properties, will effectively reduce markers for oxidative stress and inflammation.

## 2 Methods

### 2.1 Search Strategy

The guidelines established by the Preferred Reporting Items for Systematic Reviews and Meta‐Analyses (PRISMA) were utilized to perform a systematic search in three databases on September 30, 2024: PubMed, Embase, and Cochrane Library. The following boolean search query was used during the search: (Watercress) AND ((((((DNA) OR (antioxidant)) OR (oxidative Stress)) OR (lipid)) OR (Inflammat*)) OR (Damage)).

### 2.2 Article Selection

Eligibility criteria and search strategy were done following the PICOT (Population, Intervention, Comparison, Outcome, Time) framework. The patient population included patients of all ages and baseline health or comorbidities. The intervention analyzed was WC supplementation. RCTs were included to compare the effects of WC with placebo or active comparators. The outcomes in this study were the effect of supplementation on antioxidant and inflammatory markers and rates of complications. Studies with at least 1 week of follow‐up were included. Exclusion criteria consisted of case reports, review articles, animal studies, (in vivo), and in vitro (cell cultures or isolated tissues/systems) studies, conference abstracts, articles not published in English, gray literature, clinical trial registries, and articles reporting no outcomes or outcomes otherwise not specified in the inclusion criteria. Although in vivo and in vitro studies provide valuable insight into the biological mechanisms of WC, they may not provide translatable clinical outcomes in human studies (RCTs). Thus, the inclusion criteria were limited to RCTs with human participants to allow for a more clinically relevant assessment of WC's effects. This review was registered in PROSPERO as CRD42024601152.

Two reviewers independently reviewed all the studies using the predetermined eligibility criteria for title/abstract and full‐text screening. If the decision was not unanimous, a third reviewer was consulted to determine final inclusion or exclusion. All included articles underwent a thorough reference search to identify any additional studies that fit the inclusion criteria of this systematic review.

### 2.3 Quality Assessment

Two independent authors utilized the Cochrane Risk of Bias tool to determine study quality, as all included studies were RCTs (Sterne et al. 2019). The tool assesses seven domains: Sequence generation, Allocation concealment, Blinding of Participants and personnel and Blinding of Outcome Assessors, Incomplete Outcome Data, Selective Outcome Reporting, and Other sources of bias. These were evaluated as “high,” “low,” or “unclear” risk of bias. Any discrepancies were resolved by rigorous re‐evaluation of the articles until an agreement was reached.

### 2.4 Data Extraction and Statistical Analysis

Study variables included the number of patients, dosage of WC, formulation of WC, mean age, mean follow‐up time, pre‐ and post‐intervention antioxidant/anti‐inflammatory outcomes, and complications. Extracted data were collected and analyzed using Google Sheets (Google Drive; Google, Mountain View, CA). If applicable and available, descriptive statistics such as mean, percentage, standard deviations, and ranges were reported. Due to significant heterogeneity in the included studies, a meta‐analysis could not be performed, although it was originally planned. Similarly, subgroup or sensitivity analyses were unable to be performed given the small number of studies and variability in the outcomes reported.

## 3 Results

### 3.1 Literature Selection

**FIGURE 1:**
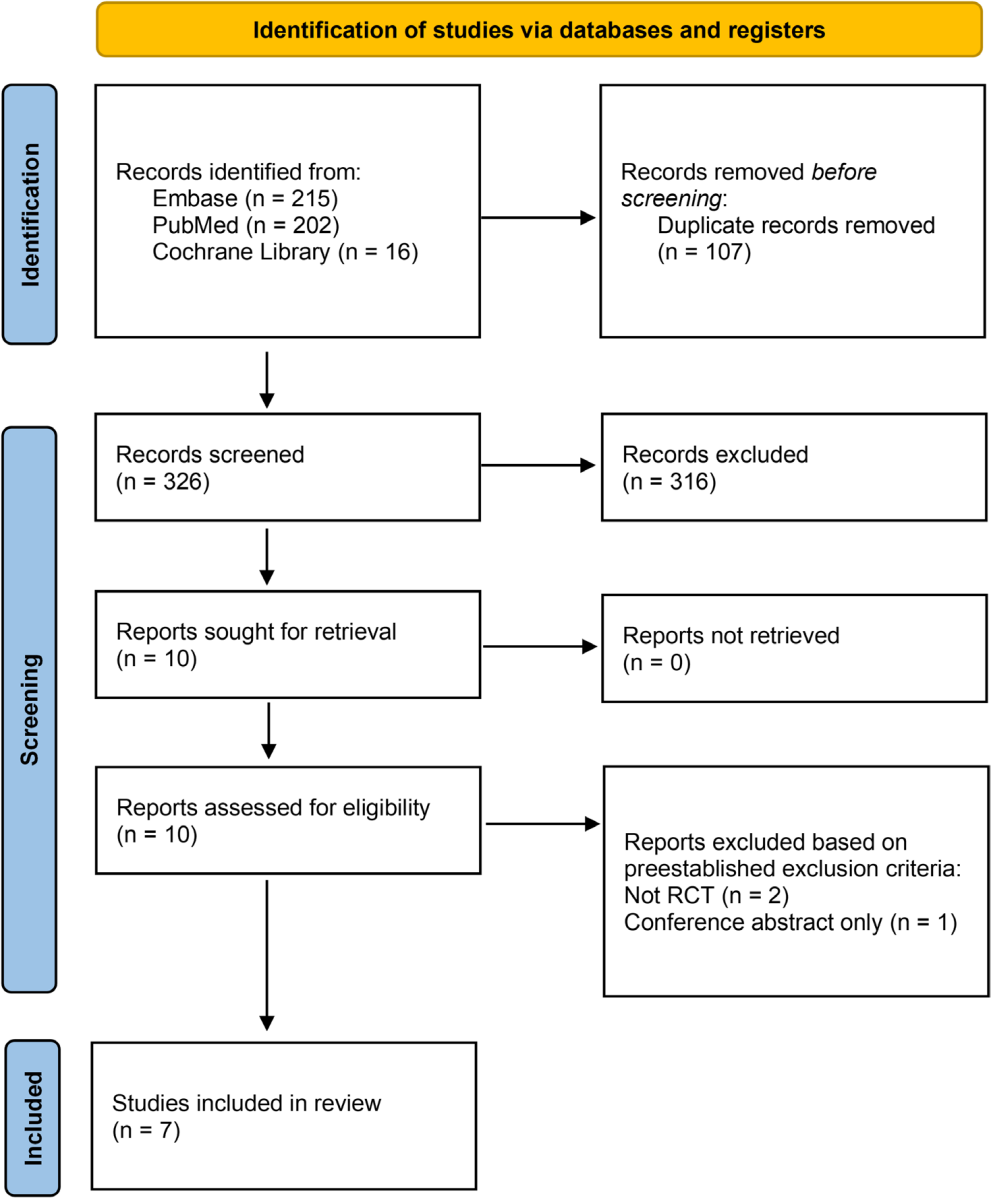
PRISMA diagram for study selection.

Using PubMed, Embase, and Cochrane Library, the initial search yielded 433 studies, of which 107 studies were identified as duplicates and removed. A total of 326 papers remained for the title and abstract screening. Ten studies were selected to undergo full‐text screening, of which 7 papers remained to be included in the systematic review (Clemente et al. 2020, 2021; Sedaghattalab, Razazan, Sadeghi, et al. 2021; Fogarty et al. 2013; Gill et al. 2007; Sedaghattalab, Razazan, Shahpari, et al. 2021; Shakerinasab et al. 2024). The screening process is detailed in Figure 1.

### 3.2 Study Characteristics

The recorded patient demographic data that were analyzed included the total number of participants, gender identification, mean age, mean follow‐up, and dosage of WC. Across the seven studies, there were a total of 302 participants (138 male, 118 female) with a mean age of 47 years (23–61 years). Sedaghattalab, Razazan, Sadeghi, et al. (2021) was the only study to not report gender demographics (Sedaghattalab, Razazan, Sadeghi, et al. 2021). The mean follow‐up time included within the studies was 39 days (21 days–60 days). Within the intervention groups, the dosage type varied based on the specific parameters. Two studies used a weight‐based dosage of 750 mg/kg/day (Clemente et al. 2020, 2021). Two studies used a dosage of 85 g/day (Fogarty et al. 2013; Gill et al. 2007). The remaining three studies used 500 mg of extract daily throughout the study (Sedaghattalab, Razazan, Sadeghi, et al. 2021; Sedaghattalab, Razazan, Shahpari, et al. 2021; Shakerinasab et al. 2024). These findings are further analyzed in Table 1.

**TABLE 1:** Study characteristics and patient demographics.

Author	Journal	Study year	Trial parameters	Number of participants	Mean age (years)	Mean follow‐up	Dosage	Adverse events
Clemente 2020	Phytotherapy Research	2020	Randomized double blind placebo controlled trial	59	40	36 days	750 mg/kg/day	NR
Clemente 2021	Phytotherapy Research	2021	Randomized double blind placebo controlled trial	34	38.8	5 weeks	750 mg/kg/day	None
Fogarty 2013	British Journal of Nutrition	2013	Randomized crossover trial	10	23	8 weeks	85 g/day	NR
Gill 2007	The American Journal of Clinical Nutrition	2007	Randomized single blind crossover trial	60	33	8 weeks	85 g/day	NR
Sedaghattalab 2021	Hindawi	2021	Randomized double blind placebo controlled trial	46	61	1 month	500 mg extract	NR
Sedaghattalab 2021#2	Biochemistry Research International	2021	Randomized double blind placebo controlled trial	45	61	1 month	500 mg extract	NR
Shakerinasab 2024	Journal of Ethnopharmacology	2024	Randomized double blind placebo controlled trial	48	59.8	1 month	500 mg extract	NR

### 3.3 Risk of Bias

Generally, the Included Studies demonstrated a low (Five Studies) to moderate (Two Studies) Risk of bias for the domains analyzed. Both studies by Clemente et al. (2020, 2021) had a low risk of bias across all domains except for other sources of bias. Fogarty and Gill both had a high risk of bias for allocation concealment and blinding of participants and personnel, as they did not report exact methods for the blinding of experiments, but other domains were low or unsure (Fogarty et al. 2013; Gill et al. 2007). Both studies by Sedaghattalab reported a low and uncertain risk of biases throughout all domains (Sedaghattalab, Razazan, Sadeghi, et al. 2021; Sedaghattalab, Razazan, Shahpari, et al. 2021). Shakerinasab demonstrated a low risk of bias for all criteria except an unsure rating for other sources of bias (Shakerinasab et al. 2024). The Cochrane risk of bias scores can be found in Table S1.

### 3.4 Antioxidants

Superoxide dismutase (SOD) was measured in three different units across five studies (Clemente et al. 2020, 2021; Sedaghattalab, Razazan, Sadeghi, et al. 2021; Gill et al. 2007; Shakerinasab et al. 2024). Sedaghattalab et al. found a significant increase (*p* < 0.001) from 29.3 ± 6.3 U/mL to 37.1 ± 8.4 U/mL within the intervention group; however, when compared to the change in controls, there was no significance (Sedaghattalab, Razazan, Shahpari, et al. 2021). In contrast, Shakerinasab et al. reported a decrease in SOD activity from 372.15 U/mL to 287.01 U/mL, which was only significant when compared to the percent change in controls (Shakerinasab et al. 2024). Values reported by Clemente et al. and Gill et al. found no significant changes with the intervention (Clemente et al. 2020, 2021; Gill et al. 2007).

Ferric‐reducing antioxidant power (FRAP) levels were recorded in two studies with mixed results (Gill et al. 2007; Shakerinasab et al. 2024). Mean pre‐ and post‐intervention values for FRAP were 1195.5 (1164–1227) to 1383.2 (1203–1563.4). Gill et al. found a slight but insignificant decrease, while Shakerinasab et al. found a significant increase within the intervention (*p* < 0.001) and when compared to placebo (*p* = 0.049) (Gill et al. 2007; Shakerinasab et al. 2024).

Glutathione peroxidase (GPx) activity was reported in two studies, with neither having a significant change with intervention or when compared to controls (Sedaghattalab, Razazan, Sadeghi, et al. 2021; Gill et al. 2007). Retinol levels were measured in two studies, with no statistically significant changes observed, but levels decreased by 0.075 μmol/L when averaged across both studies (Fogarty et al. 2013; Gill et al. 2007). B‐carotene levels were reported in two studies, with Fogarty et al. having no difference in pre‐ and post‐intervention (Fogarty et al. 2013; Gill et al. 2007). However, Gill et al. found that β‐carotene levels had a significant increase (*p* < 0.001) after intervention from 0.33 ± 0.19 μmol/L to 0.43 ± 0.26 μmol/L when compared to the control phase (Gill et al. 2007). Despite a‐tocopherol not having a significant change in Gill et al. Fogarty et al. found that the main lipid‐soluble antioxidants, including a‐tocopherol, y‐tocopherol, and xanthophyll, were all significantly increased (*p* < 0.05) following WC supplementation when compared to controls (Fogarty et al. 2013; Gill et al. 2007). These variables are further outlined in Table 2.

**TABLE 2:** Anti‐oxidant parameters reported across the studies.

Marker	Study	Post‐treatment placebo	Post‐treatment watercress	Post‐treatment watercress vs. placebo
SOD	Clemente 2020	NS	NS	NS
	Clemente 2021	NS	NS	NS
	Sedaghattalab 2021	**↑ *p* < 0.001**	**↑ *p* < 0.001**	NS
	Shakerinasab 2023	NS	NS	***p* < 0.001**
	Gill 2007	NS	NS	NS
FRAP	Gill 2007	NS	NS	NS
	Shakerinasab 2023	**↑ *p* = 0.015**	**↑ *p* < 0.001**	***p* = 0.049**
GPX	Gill 2007	NS	NS	NS
	Sedaghattalab 2021	NS	NS	NS
Retinol Vit A	Gill 2007	NS	NS	NS
	Fogarty 2013	NS	NS	NS
A‐tocopherol	Gill 2007	NS	NS	NS
	Fogarty 2013	**↓ *p* < 0.05**	**↑ *p* < 0.05**	***p* < 0.05**
B‐carotene	Gill 2007	NS	NS	***p* < 0.001**
	Fogarty 2013	NS	NS	NS

*Note:* Bold values indicate significant results of *p* < 0.05.

### 3.5 Oxidative Stress

Protein carbonyls (PC) were measured in four studies, with significance reported in two (Clemente et al. 2020; Fogarty et al. 2013; Sedaghattalab, Razazan, Shahpari, et al. 2021; Shakerinasab et al. 2024). Sedaghattalab et al. found that PC formation significantly decreased (*p* = 0.006) from 20.33 ± 4.40 μmol/mg protein to 15.06 ± 6.41 μmol/mg protein after 1 month of intervention. There was also a significant difference when compared to the control (*p* = 0.001) (Sedaghattalab, Razazan, Sadeghi, et al. 2021). Shakerinasab et al. found an insignificant decrease in PC from 9.08 (7.80–10.95) μmol/L to 5.60 (4.45–6.54) μmol/L. However, there was a statistically significant change (*p* < 0.001) when the percent change between placebo and WC was compared (Shakerinasab et al. 2024).

Thiobarbituric acid reactive substances (TBARS) were significantly lowered (*p* < 0.05) in both studies that analyzed lipid peroxidase (LPO). Across both studies by Clemente et al. the pre‐ and post‐intervention value means were 0.0170 ± 0.0008 nmol/mL to 0.0139 ± 0.0009 nmol/mL (Clemente et al. 2020, 2021). There was also a significant difference within the intervention group and when compared to healthy controls and placebo (*p* < 0.05) (Clemente et al. 2020).

There was a significant change in both papers that analyzed malondialdehyde (MDA). The mean values decreased from 1.83 mmol/L to 1.00 mmol/L (Sedaghattalab, Razazan, Shahpari, et al. 2021; Shakerinasab et al. 2024). Both Sedaghattalab et al. and Shakerinasab et al. found significance in the percent change between the placebo and intervention groups, which were *p* < 0.001 and *p* = 0.01, respectively; however, only Sedaghattalab et al. found a significant decrease in the intervention group (*p* < 0.001) (Sedaghattalab, Razazan, Sadeghi, et al. 2021; Shakerinasab et al. 2024).

Nitric oxide metabolites were reported in two studies with mean pre‐ and post‐intervention values of 24.99 μmol/L to 23.89 μmol/L. However, only Shakerinasab et al. found significance between the percent change of the placebo and treatment groups, though both studies found no significance in the change within the treatment groups (Sedaghattalab, Razazan, Shahpari, et al. 2021; Shakerinasab et al. 2024).

Total thiol (T‐SH) was reported in two studies with a mean change of 12.64 μmol/L to 10.11 μmol/L (Sedaghattalab, Razazan, Sadeghi, et al. 2021; Sedaghattalab, Razazan, Shahpari, et al. 2021). The decrease in Sedaghattalab et al. was found to be significant; however, there was no difference in the percent change between placebo and WC supplementation (Sedaghattalab, Razazan, Sadeghi, et al. 2021). Catalase (CAT) activity was measured in three studies, with no significant changes (Clemente et al. 2020, 2021; Shakerinasab et al. 2024). These variables are further outlined in Table 3.

**TABLE 3:** Anti‐inflammatory markers reported across studies.

Marker	Study	Post‐treatment placebo	Post‐treatment watercress	Post‐treatment watercress vs. placebo
CAT	Clemente 2020	NS	NS	NS
	Clemente 2021	NS	NS	NS
	Shakerinasab 2023	NS	NS	NS
Protein carbonyls	Fogarty 2013	NS	NS	NS
	Sedaghattalab 2021#2	**↑ *p* = 0.022**	**↓ *p* = 0.006**	***p* = 0.001**
	Shakerinasab 2023	NS	NS	***p* < 0.001**
	Clemente 2020	NS	NS	NS
TBARS	Clemente 2020	NS	**↓ *p* < 0.05**	NS
	Clemente 2021	NS	NS	***p* < 0.05**
MDA	Sedaghattalab 2021	**↓ *p* < 0.001**	**↓ *p* < 0.001**	***p* < 0.001**
	Shakerinasab 2023	NS	NS	***p* = 0.01**
NO	Sedaghattalab 2021#2	NS	NS	NS
	Shakerinasab 2023	**↓ *p* = 0.001**	NS	***p* = 0.048**
T‐SH	Shakerinasab 2023	NS	NS	NS
	Sedaghattalab 2021	**↓ *p* < 0.001**	**↓ *p* < 0.001**	NS

*Note:* Bold values indicate significant results of *p* < 0.05.

Gill et al. measured DNA stability based on the mean difference between basal DNA damage and basal DNA damage plus oxidative‐induced DNA damage (Gill et al. 2007). After 8 weeks of supplementation with WC and compared to placebo, WC had caused a significant difference of 17% (*p* = 0.03) and 22.9% (*p* = 0.002) in both measurements, respectively.

### 3.6 Inflammatory Markers

TNF‐α was measured in two studies, with a slight decrease reported in both. TNF‐α mean concentration decreased from 10.2 pg/mL to 9.98 pg/mL after WC intervention; however, there were no significant changes in the intervention group or placebo (Sedaghattalab, Razazan, Shahpari, et al. 2021; Shakerinasab et al. 2024).

Other inflammatory markers that were reported by individual studies include interleukin‐1 (IL‐1), IL‐6, and C‐reactive protein (CRP). IL‐1 increased from 0.84 (0.40–1.28) pg/mL to 1.28 (0.62–1.95) pg/mL, IL‐6 decreased from 60.10 (55.99–73.10) pg/mL to 55.21 (53.39–60.48) pg/mL, and CRP decreased from 8953.3 ± 5588.06 to 7249.86 ± 5091.62, all of which were significant for WC (*p* = 0.029, *p* = 0.05, *p* = 0.007, respectively) (Sedaghattalab, Razazan, Sadeghi, et al. 2021; Shakerinasab et al. 2024). However, only IL‐1 was found to be significant compared to placebo (*p* = 0.004) (Shakerinasab et al. 2024). These values are further outlined in Table 4.

**TABLE 4:** Inflammatory markers reported across studies.

Marker	Study	Post‐treatment placebo	Post‐treatment watercress	Post‐treatment watercress vs. placebo
TNF‐a	Sedaghattalab 2021#2	NS	NS	NS
	Shakerinasab 2023	NS	NS	NS
IL‐6	Sedaghattalab 2021#2	NS	**↓ *p* = 0.050**	NS
IL‐1	Shakerinasab 2023	NS	**↑ *p* = 0.029**	***p* = 0.004**
CRP	Sedaghattalab 2021#2	NS	**↓ *p* = 0.007**	NS

*Note:* Bold values indicate significant results of *p* < 0.05.

### 3.7 Lipids

Total cholesterol and HDL were measured in three studies without significant changes (Clemente et al. 2021; Sedaghattalab, Razazan, Shahpari, et al. 2021; Gill et al. 2007). Only two of the three studies reported values with a mean decrease of total cholesterol from 164 mg/dL to 159 mg/dL and HDL from 47.3 to 46.9 mg/dL. Triglyceride levels also did not have any significance from pre‐ and post‐intervention, with a mean decrease of 107 mg/dL to 105 mg/dL; however, Sedaghattalab et al. did note a significant increase in triglyceride levels in the placebo group (Sedaghattalab, Razazan, Sadeghi, et al. 2021).

LDL was also reported in the same three studies (Clemente et al. 2021; Sedaghattalab, Razazan, Shahpari, et al. 2021; Gill et al. 2007). Despite not having reported values in their study, Clemente et al. found there to be a significant difference in LDL levels post‐intervention when compared to placebo (*p* < 0.01) (Clemente et al. 2021). The other two studies showed a slight decrease in LDL from 92 mg/dL to 89 mg/dL with no significance after intervention (Sedaghattalab, Razazan, Sadeghi, et al. 2021; Gill et al. 2007). However, Sedaghattalab et al. found that the placebo group had a significant increase in LDL after 1 month (*p* < 0.04) (Sedaghattalab, Razazan, Shahpari, et al. 2021). These variables are further outlined in Table 5. The full breakdown of the data and outcome variables can be found in Appendices S1–S4.

**TABLE 5:** Lipid markers reported across studies.

Marker	Study	Post‐treatment placebo	Post‐treatment watercress	Post‐treatment watercress vs. placebo
Triglycerides	Clemente 2021	—	—	NS
	Gill 2007 $	NS	NS	NS
	Sedaghattalab 2021	**↑ *p* < 0.01**	NS	NS
Total cholesterol	Clemente 2021	—	—	NS
	Gill 2007 $	NS	NS	NS
	Sedaghattalab 2021	NS	NS	NS
HDL	Clemente 2021	—	—	NS
	Gill 2007 $	NS	NS	NS
	Sedaghattalab 2021	NS	NS	NS
LDL	Clemente 2021	—	—	***p* < 0.01**
	Gill 2007 $	NS	NS	NS
	Sedaghattalab 2021	**↑ *p* < 0.04**	NS	NS

*Note:* Bold values indicate significant results of *p* < 0.05.

## 4 Discussion

This systematic review evaluated seven RCTs, utilizing WC and its anti‐oxidative and anti‐inflammatory properties. Through analysis of pre‐ and post‐intervention outcomes, laboratory values, and rates of adverse events, WC demonstrates an improvement in anti‐oxidative and anti‐inflammatory markers, laboratory values, and a strong safety profile.

**FIGURE 2:**
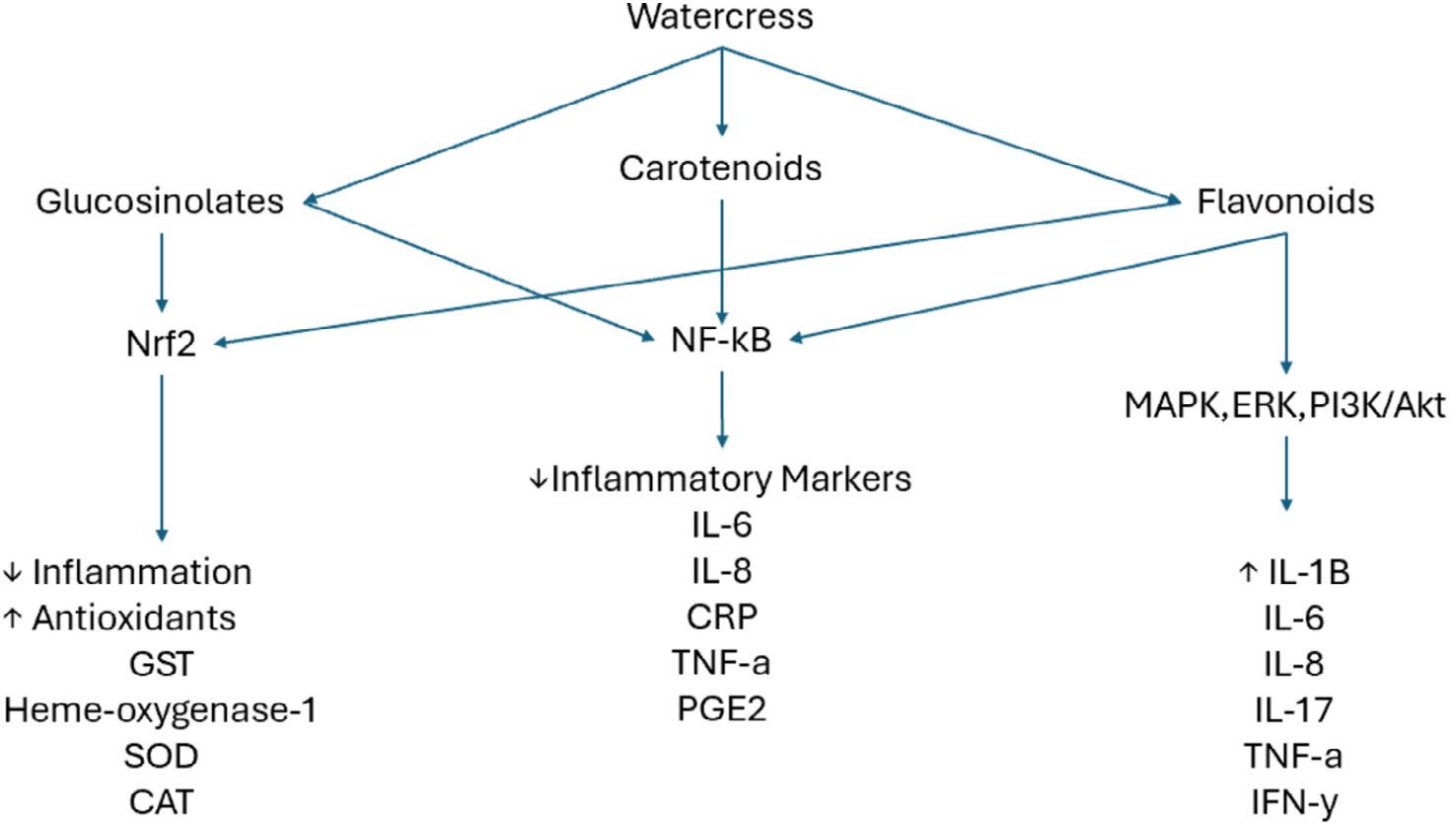
Antioxidant and anti‐inflammatory pathways affected by watercress.

Watercress contains one of the highest concentrations of glucosinolates and carotenoids from any vegetable, which exerts its anti‐oxidant and anti‐inflammatory effects through several molecular pathways (Shree et al. 2022). Glucosinolates (GLS), as a precursor, are converted into the bioactive compound phenethyl isothiocyanate (PEITC) upon tissue disruption due to the action of thioglucosidase myrosinase (Peluso et al. 2013). In animal and human studies, PEITC possesses anticarcinogenic properties by inducing apoptosis and disrupting cancer cell proliferation. PEITC was also shown to reduce inflammation by increasing the activity of the nuclear factor erythroid 2‐related factor 2 (Nrf2) pathway, which increases antioxidant pathways and reduces free radical production and inflammation (Walsh et al. 2023; Clemente et al. 2021). Carotenoids such as lutein and beta‐carotene have antioxidant, anti‐inflammatory, and anti‐apoptotic properties (Kaulmann and Bohn 2014). Through in vitro, animal, and human studies, these molecules enact their effects by influencing intracellular signaling cascades, thereby affecting gene and protein expression (Kaulmann and Bohn 2014). Most notably, carotenoids inhibit the nuclear factor kB (NF‐kB) pathway, which inhibits the downstream production of inflammatory molecules such as CRP, TNF‐α, IL‐6, interleukin‐8 (IL‐8), and prostaglandin E2 (PGE2) (Kaulmann and Bohn 2014). Its anti‐oxidant effects are mainly influenced by the Nrf2 pathway, which activates antioxidants such as glutathione‐S‐transferases (GST) (Kaulmann and Bohn 2014). Flavonoids such as quercetin, kaempferol, genistein, apigenin, and epigallocatechin 3‐gallate are also abundant in WC (Al‐Khayri et al. 2022). Flavonoids reduce inflammation and oxidative stress by modulating the NF‐kB, mitogen‐activated protein kinase (MAPK), extracellular signal‐regulated kinase (ERK), and phosphoinositide 3 kinase (PI3K)/Akt pathways (Al‐Khayri et al. 2022). Aside from reducing inflammatory cytokines such as IL‐1B, IL‐6, IL‐8, interleukin‐17 (IL‐17), TNF‐a, and interferon‐gamma (IFN‐y), flavonoids also reduce enzymes such as cyclooxygenase (COX), lipoxygenase (LOX), glucuronidase, lysozyme, and inducible nitric oxide synthase (iNOS) (Al‐Khayri et al. 2022). These enzymes enhance the Nrf2 and AMPK pathways and increase levels of antioxidant enzymes such as GST, heme‐oxygenase‐1, SOD, and CAT, which regulate cell viability (Al‐Khayri et al. 2022). These pathways which WC's effect is illustrated in Figure 2.

These antioxidant and anti‐inflammatory effects are consistent with findings in other popular cruciferous vegetables such as broccoli, cabbage, and kale through overlapping pathways (Syed et al. 2023; Zhang et al. 2024). Sulforaphane in broccoli reduces inflammation through inhibition of IL‐6 and TNF‐α and modulates inflammatory pathways of NF‐kB, Nrf2, and MAPK (Syed et al. 2023). Furthermore, broccoli contains vitamins C, E, β‐carotene, and other flavonoids such as Quercetin that regulate oxidative stress (Syed et al. 2023). Mouse models have demonstrated broccoli extracts' ability to reduce liver enzymes and inflammatory cytokines while increasing levels of antioxidant enzyme activity (Mao et al. 2023). Additionally, this was seen in lipopolysaccharide‐induced lung injury mouse models as well by activating the Nrf2/antioxidant response element (ARE) pathway, which suppressed the NF‐kB inflammatory response (decreased IL‐6, TNF‐α) (Qi et al. 2016). Anthocyanins (ACNs) present in broccoli, cabbage, and kale exert potent antioxidant and anti‐inflammatory effects via the aforementioned pathways as well (Chatupos et al. 2024). The synergistic effects of polyphenols and other bioactive compounds in cruciferous vegetables are generally explained through the “biochemical scavenger theory,” where free radicals are neutralized by the polyphenol compounds, preventing further reactions (Lučić et al. 2023).

### 4.1 Anti‐Oxidative Effects of Watercress

Oxidative stress is induced due to the generation of reactive oxygen species (ROS), which causes a variety of harmful effects that lead to cell death (Keser and Saygideger 2010). Increased levels of SOD are responsible for the efficient breakdown of ROS and its metabolites. CAT, another enzyme involved in removing toxic peroxides, performs this function by reducing hydrogen peroxide (H_2_O_2_) into oxygen (O_2_) and water (H_2_O) (Keser and Saygideger 2010). Decreased levels of MDA demonstrate reduced levels of oxidative stress, as MDA is a marker for free radical‐mediated injury (Keser and Saygideger 2010). Superoxide (O_2_−) radicals are generated during inflammatory conditions and are known to cause direct oxidative DNA damage or be dismutated to H_2_O_2_ (Misiaszek et al. 2004). H_2_O_2_ readily diffuses across the cell membrane, which can form hydroxyl radicals (OH−), resulting in single and double‐stranded DNA breaks and base pair modifications (Fogarty et al. 2013). It has also been proposed to be a direct mediator of DNA damage by disrupting repair pathways. O_2_− and OH− can also cause lipid peroxidation, generating lipid hydroperoxides (LOOH), leading to further DNA damage (Fogarty et al. 2013). Polyunsaturated fatty acid breakdown products, such as mutagenic compounds and lipid peroxidation intermediates, can directly damage DNA, while alkoxyl free radicals may lead to lipid peroxidation (Fogarty et al. 2013). Fogarty et al. demonstrated that both acute (2 h) and chronic (8 weeks) supplementation of WC decreased lipid peroxidation, H_2_O_2_ levels, and LOOH while markedly increasing lipid‐soluble antioxidant levels (Fogarty et al. 2013). The high lipid‐soluble antioxidant levels have been postulated to be a mechanism for DNA stabilization (Gill et al. 2007). In rat models, WC attenuated liver damage by decreasing hydroxyproline levels (Sadeghi et al. 2019). Additionally, it reduced histopathological indexes and oxidative stress by preventing protein oxidation and increased the activity of GPx, enhancing free‐radical scavenging (Sadeghi et al. 2019). Interestingly, upon induced oxidative stress by cyclophosphamide, WC reversed its effects on LPO, SOD, CAT, and reduced to oxidized glutathione (GSH) ratio (Casanova et al. 2017).

### 4.2 Anti‐Tumor Effects of Watercress

WC exhibits anti‐tumor activity due to its modulation of axes that control cell cycle progression, apoptosis, and MAPK (Panahi Kokhdan et al. 2021). Isothiocyanate (ITC), a breakdown product of glucosinolates, has been characterized as playing a key role in carcinogenesis, with strong tumor‐protective effects shown in experimental and animal models (Keser and Saygideger 2010; Sakao et al. 2015). In cell lines, it was shown that WC protected against DNA damage in colon cancer HT29 cells (induced by H_2_O_2_, 4‐hydroxy nonenal, fecal water), hepatic hepg2 cells (induced by benzo (a) pyrene), MDA‐MB‐231 breast cancer cell line (matrix metalloproteinase‐9), and reduced the survival rate of cancerous HeLa cells (Panahi Kokhdan et al. 2021; Boyd et al. 2006; Rose et al. 2005). In human studies, 56.8 g of WC for 3 days attenuated the activation of 4‐(methylnitrosamino)‐1‐(3‐pyridyl)‐1‐butanone, a prevalent carcinogen in tobacco (Giallourou et al. 2019). A 2018 study also detailed the potential prophylactic impact of WC and PEITC during radiotherapy by modulating cellular bioenergetics and increasing levels of glutathione (Giallourou et al. 2019). Watercress protected non‐tumorigenic breast cells from radiation‐induced damage while PEITC enhanced the cancer‐killing effectiveness of radiation by increasing breast cancer cell sensitivity to ionizing radiation (Giallourou et al. 2019). Carotenoids also demonstrate anti‐tumor activity for various cancers such as breast, colorectal, lung, and prostate cancer (Saini et al. 2020). There is also a synergistic action in combination with conventional chemotherapeutic agents and increased sensitization of tumor cells to conventional treatment (Saini et al. 2020). However, carotenoids exhibit poor solubility and bioavailability (Koklesova et al. 2020).

### 4.3 Anti‐Inflammatory Effects of Watercress

Among the ITCs, PEITC and 8‐methylsulphinyloctyl (MSO) are two derivative phytochemical components in WC (Rose et al. 2005). In vitro studies demonstrate that both molecules reduce proinflammatory mediators such as COX‐2 and iNOS by modulating the NF‐kB pathway, which decreases nitrite and PGE2 levels (Rose et al. 2005). Sadeghi et al. recommended that topical and systemic WC could be used as an anti‐inflammatory agent for inflammatory diseases (Sadeghi et al. 2014). Furthermore, another study supported the topical and systemic anti‐inflammatory effects of WC in rats for two chronic inflammatory models (carrageenan and formalin) (Mostafazadeh et al. 2022). Another study demonstrated WC's (50, 100, 200 mg/kg/d) role in attenuating oxidative stress and inflammation for gentamicin‐induced nephrotoxicity through decreased nephrotoxic markers (ROS, GSH, LPO, PC) and oxidative stress/inflammation markers (NO and TNF‐a) (Shahani et al. 2017). These nephroprotective findings were supported in a study with hemodialysis patients where a significant reduction was found in the PCO levels with the WC group, as well as compared to the control group (Sedaghattalab, Razazan, Sadeghi, et al. 2021). There was also a significant decrease in CRP and IL‐6 in the WC group, but not when compared to placebo (Sedaghattalab, Razazan, Shahpari, et al. 2021). Recent studies postulate that WC can exhibit hepatoprotective effects as well. Azarmehr et al. investigated its effects on acetaminophen (APAP)‐induced hepatotoxicity in rats (Azarmehr et al. 2019). They found a significant decrease in aspartate aminotransferase (AST) and an increase in T‐SH and GPx activity compared to the APAP group (Azarmehr et al. 2019). In bile‐duct‐induced cholestatic rats, WC markedly decreased liver PCO, hydroxyproline levels, and histopathologic indexes while increasing Gpx levels (Sadeghi et al. 2019). These findings suggest that WC may be a possible therapeutic agent for nephrotoxicity and hepatotoxicity.

### 4.4 Limitations

The results from this study must be taken into consideration, along with its limitations. First, there was heterogeneity within the study designs, comparator groups, dosage of WC, patient demographics, short follow‐up times, and variability in outcome reporting/units. The small number of available RCTs and variability in outcome reporting prevent a meta‐analysis from being performed, limiting the calculation of pooled effects of WC and subsequently the statistical power to generalize these conclusions for WC. Additionally, subgroup and sensitivity analyses were unable to be performed given the small number of studies and outcome measures reported, which underscores the need for more standardized RCTs in this area. The high or unclear risk of bias in several studies also limits the generalizability of these findings. Paradoxical/inconsistent findings for markers such as IL‐1 highlight the discrepancy that may be seen due to the heterogeneity of WC formulation, dosage, matrix interaction with diet, baseline patient demographics, or laboratory measurement variability. Future studies with follow‐up durations greater than 12 weeks should aim to standardize the patient demographics and inflammatory and oxidative markers evaluated (IL‐1, IL‐6, TNF‐a, CRP, SOD, PC, TBARS, MDA, and NO) to better capture whether these biochemical effects are sustained. Additionally, the formulations/composition of WC and the control/active comparator groups should also be standardized to reduce heterogeneity. Fresh watercress and watercress extracts may differ in bioavailability and concentrations of bioactive compounds depending on growing conditions or preparations. Thus, these were grouped in this review to allow a broader understanding of WC's effects despite the added heterogeneity. Comparator group variations with confounding factors such as dietary advice, background diets, or co‐interventions would reduce inter‐study comparability and increase the heterogeneity in outcomes. Comparisons between WC and other vegetables should also be evaluated in clinical trials to determine which vegetable provides the greatest benefits. Second, the dietary levels of antioxidants such as flavonoids and carotenoids were not accounted for, which can affect the results in the studies analyzed. Third, the effects of WC may differ depending on the species of WC, its growing conditions, composition, doses used, and total follow‐up time/exposure to the supplement. Fourth, the follow‐up time was relatively short. Thus, mid‐ to long‐term studies are necessary to better support the findings in this study. Fifth, gray literature and clinical trial registries were not searched, which may limit these results and potential publication bias. Given the limited number of RCTs available, a broader inclusion criterion was used to ensure adequate data inclusion.

## 5 Conclusion

In this systematic review, we aimed to more thoroughly assess the anti‐inflammatory effects of watercress by analyzing studies that investigated inflammatory mediators in human populations following watercress administration. An analysis of 7 RCTs found that watercress had a positive effect on antioxidant and anti‐inflammatory markers, such as SOD, PC, TBARS, MDA, and NO. Although the studies assessed had relatively short follow‐up periods and were heterogeneous in terms of their design, this systematic review may serve as a basis for future studies to assess further how watercress may be adapted to the treatment of inflammatory conditions.

## Author Contributions

**Jimmy Wen:** conceptualization (equal), data curation (equal), formal analysis (equal), investigation (equal), methodology (equal), project administration (equal), resources (equal), supervision (equal), validation (equal), visualization (equal), writing – original draft (equal), writing – review and editing (equal). **Muhammad Karabala:** conceptualization (equal), data curation (equal), formal analysis (equal), investigation (equal), methodology (equal), validation (equal), visualization (equal), writing – original draft (equal), writing – review and editing (equal). **Zohaer Muttalib:** conceptualization (equal), data curation (equal), formal analysis (equal), investigation (equal), methodology (equal), validation (equal), writing – original draft (equal), writing – review and editing (equal). **Burhaan Syed:** conceptualization (equal), data curation (equal), formal analysis (equal), investigation (equal), methodology (equal), writing – original draft (equal), writing – review and editing (equal). **Ramy Khalil:** conceptualization (equal), data curation (equal), investigation (equal), software (equal), validation (equal), writing – original draft (equal), writing – review and editing (equal). **Daniel Razick:** conceptualization (equal), data curation (equal), investigation (equal), project administration (equal), writing – original draft (equal), writing – review and editing (equal). **Adam Razick:** conceptualization (equal), data curation (equal), project administration (equal), visualization (equal), writing – original draft (equal), writing – review and editing (equal). **David Pai:** conceptualization (equal), project administration (equal), supervision (equal), writing – original draft (equal), writing – review and editing (equal).

## Ethics Statement

The authors have nothing to report.

## Consent

The authors have nothing to report.

## Conflicts of Interest

The authors declare no conflicts of interest.

## Supporting information


Appendices S1–S4

Table S1

## Data Availability

The datasets used and/or analyzed in the current study are available upon reasonable request. Please contact J.W. to request data from the study.
