# Supplementary material for: Evaluating the Anti‐Oxidant and Anti‐Inflammatory Properties of Watercress Supplementation at Short‐Term Follow‐Up: A Systematic Review of Randomized Controlled Trials
Source: Food Sci Nutr. 2025 Jun 5;13(6):e70407. doi: 10.1002/fsn3.70407 (PMC12141087; doi:10.1002/fsn3.70407)
Supplement: Supplementary file 1 — Appendices S1–S4 [file FSN3-13-e70407-s001.docx]

| Marker | Study | Placebo Pre-Treatment | Placebo Post-Treatment | Placebo Significance | Watercress Pre-treatment | Watercress Post-Treatment | Watercress Sig | Significance Watercress vs Placebo |
| --- | --- | --- | --- | --- | --- | --- | --- | --- |
| SOD | Clemente 2020 | 270.8 ± 54.14 mmol/min/mL | 292.5 ± 59.50 mmol/min/mL | NR | 311.4 ± 37.65 mmol/min/mL | 174.4 ± 23.25 mmol/min/mL | NR | Not significant |
|  | Clemente 2021 | 249.8 ± 55.99 mmol/min/mL | 226.7 ± 41.91 mmol/min/mL | NR | 304.3 ± 55.66 mmol/min/mL | 165.4 ± 31.34 mmol/min/mL | NR | Not significant |
|  | Sedaghattalab 2021 *** | 28.3 ± 8.1 U/mL | 33.8 ± 5.7 U/mL | **p<0.001** | 29.3 ± 6.3 U/mL | 37.1 ± 8.4 U/mL | **p<0.001** | p<0.32 |
|  | Shakerinasab 2023 **** | 372.15 (355.68 - 380.52) U/mL | 297.80 (278.02 - 314.99) U/mL | p=0.468 | 372.15 (355.68–380.52) u/mL | 287.01 (278.02–295.77) u/mL | p=0.575 | **p<0.001** |
|  | Gill 2007 | 1291 ± 240 U/g hemoglobin | 1304 ± 247 U/g hemoglobin | NR | 1283 ± 243 U/g hemoglobin | 1297 ± 239 U/g hemoglobin | NR | NR |
| FRAP | Gill 2007 | 1230 ± 240 umol/L | 1169 ± 194 umol/L | NR | 1227 ± 239 umol/L | 1203 ± 184.24 umol/L | NR | NR |
|  | Shakerinasab 2023 *** | 1067.75 (914.93 - 1191.50) umol/L | 1228.37 (1061.18 - 1472.43) umol/L | **p=0.015** | 1164.62 (960.25–1278.06) umol/L | 1573.37 (1390.87–1715.25) umol/L | **p<0.001** | **p=0.049** |
| GPX | Gill 2007 | 43.74 ± 11.54 u/1g hemoglobin | 44 ± 11.95 u/1g hemoglobin | NR | 43.95 ± 11.38 u/1g hemoglobin | 44.66 ± 12.01 u/1g hemoglobin | NR | NR |
|  | Sedaghattalab 2021 | 223 ± 115 U/mL | 252.9 ± 107.8 U/mL | p<0.17 | 164.2 ± 103.6 u/mL | 173.3 ± 134.4 u/mL | p<0.56 | p<0.54 |
| Retinol Vit A | Gill 2007 | 1.89 ± 0.43 umol/L | 1.79 ± 0.37 umol/L | NR | 1.89 ± 0.39 umol/L | 1.84 ± 0.36 umol/L | NR | NR |
|  | Fogarty 2013 | 1.9 ± 0.2 mmol/L | 1.9 ± 0.4 mmol/L | NR | 2.1 ± 0.4 mmol/L | 2.0 ± 0.3 mmol/l | NR | NR |
| A-tocopherol | Gill 2007 | 27.28 ± 6.71 umol/L | 26.31 ± 5.37 umol/L | NR | 27.29 ± 6.23 umol/L | 26.61 ± 6.13 umol/L | NR | Not significant |
|  | Fogarty 2013 *** | 122 ± 10 mmol/L | 114 ± 7 mmol/L | **p<0.05** | 118 ± 21 mmol/L | 146 ± 35 mmol/L | **p<0.05** | **p<0.05** |
| B-carotene | Gill 2007 *** | 0.32 ± 0.21 umol/L | 0.33 ± 0.2 umol/L | NR | 0.33 ± 0.19 umol/L | 0.43 ± 0.26 umol/L | NR | p<0.001 |
|  | Fogarty 2013 | 0.7 ± 0.01 mmol/L | 0.8 ± 0.04 mmol/L | NR | 1.1 ± 0.02 mmol/L | 1.1 ± 0.02 mmol/L | NR | NR |

**Appendix S1: Anti-oxidant parameters reported across the studies**

| Marker | Study | Placebo Pre-Treatment | Placebo Post-Treatment | Placebo Significance | Watercress Pre-treatment | Watercress Post-treatment | Watercress Significance | Significance |
| --- | --- | --- | --- | --- | --- | --- | --- | --- |
| CAT | Clemente 2020 | 11.38 ± 1.534 mmol/min/mL | 11.8 ± 1.313 mmol/min/mL | NR | 13.17 ± 0.995 mmol/min/mL | 8.981 ± 0.666 mmol/min/mL | NR | No significance |
|  | Clemente 2021 | 11.17 ± 1.495 mmol/min/mL | 11.11 ± 1.201 mmol/min/mL | NR | 11.56 ± 0.957 mmol/min/mL | 9.32 ± 0.837 mmol/min/mL | NR | No significance |
|  | Shakerinasab 2023 | 1.65 (0.52 - 3.64) U/mL | 0.81 (0.28 - 1.56) U/mL | p=0.892 | 2.64 (1.49 - 4.41) U/mL | 1.31 (0.62 - 2.29) U/mL | p=0.63 | p=0.605 |
| Protein Carbonyls | Fogarty 2013 | 0.9 ± 0.1 mg/total protein | 0.9 ± 0.2 mg/total protein | NR | 0.9 ± 0.1 mg/total protein | 0.9 ± 0.1 mg/total protein | NR | NR |
|  | Sedaghattalab 2021#2*** | 22.19 ± 4.26 umol/mg protein | 26.46 ± 7.72 umol/mg protein | **p=0.022** | 20.33 ± 4.40 umol/mg protein | 15.06 ± 6.41 umol/mg protein | **p=0.006** | **p=0.001** |
|  | Shakerinasab 2023*** | 8.65 (7.54 - 10.47) umol/L | 8.47 (6.32 - 9.95) umol/L | p=0.333 | 9.08 (7.80 - 10.95) umol/L | 5.60 (4.45 - 6.54) umol/L | p=0.701 | **p<0.001** |
|  | Clemente 2020 | 0.0506 ± 0.0053 mol/mg | 0.0503 ± 0.0051 mol/mg | NR | 0.0495 ± 0.0042 mol/mg | 0.0359 ± 0.0031 mol/mg | NR | No significance |
| TBARS | Clemente 2020*** | 0.0138 ± 0.0006 nmol/mL | 0.0140 ± 0.0006 nmol/L | NR | 0.0163 ± 0.0007 nmol/mL | 0.0131 ± 0.0008 nmol/mL | **p<0.05 compared to control** | NR |
|  | Clemente 2021 | 0.0122 ± 0.0006 nmol/mL | 0.0111 ± 0.0010 nmol/mL | NR | 0.0176 ± 0.0009 nmol/mL | 0.0146 ± 0.0009 nmol/mL | NR | **p<0.05 compared to placebo** |
| MDA | Sedaghattalab 2021 | 1.5 ± 0.14 mmol/L | 0.67 ± 0.22 mmol/L | **p<0.001** | 1.6 ± 0.13 mmol/L | 0.42 ± 0.27 mmol/L | **p<0.001** | **p<0.001** |
|  | Shakerinasab 2023 | 1.96 (1.66-2.66) umol/L | 2.16 (1.55 - 2.73) umol/L | p=0.694 | 2.05 (1.49 - 2.68) umol/L | 1.58 (1.13 - 1.88) umol/L | p=0.555 | **p=0.01** |
| NO | Sedaghattalab 2021#2 | 9.12 ± 5.88 umol/L | 14.25 ± 9.41 umol/L | p=0.60 | 12.25 ± 7.66 umol/L | 15.00 ± 12.10 umol/L | p=0.312 | p=0.525 |
|  | Shakerinasab 2023 | 39.77 (31.43 - 55.03) umol/L | 23.30 (17.79 - 25.88) | **p=0.001** | 37.79 (28.30 - 60.22) umol/L | 8.89 (6.50 - 15.58) umol/L | p=0.232 | **p=0.048** |
| T-SH | Shakerinasab 2023 | 12.50 (11.67 - 14.30) umol/L | 15.25 (11.87 - 18.40) umol/L | p=0.376 | 12.17 (10.17 - 14.19) umol/L | 12.82 (9.59 - 16.08) umol/L | p=0.329 | p=0.488 |
|  | Sedaghattalab 2021 | 10.1 ± 4.1 mmol/L | 5.6 ± 3.2 mmol/L | **p<0.001** | 13.1 ± 5.3 mmol/L | 7.4 ± 4.3 mmol/L | **p<0.001** | p<0.323 |

**Appendix S2: Anti-inflammatory markers reported across studies**

| Marker | Study | Placebo Pre-Treatment | Placebo Post-Treatment | Placebo Significance | Pre-treatment Watercress | Post-treatment Watercress | Watercress Sig | Significance |
| --- | --- | --- | --- | --- | --- | --- | --- | --- |
| TNF-a | Sedaghattalab 2021#2 | 15.95 (15.17 - 16.66) pg/mL | 15.87 (14.75 - 16.22) pg/mL | p=0.330 | 15.96 (15.16 - 16.66) pg/mL | 15.94 (15.44 - 16.53) pg/mL | p=0.615 | p=0.276 |
|  | Shakerinasab 2023 | 4.40 (4.09 - 5.01) pg/mL | 4.16 (2.78 - 4.55) pg/mL | p=0.446 | 4.40 (4.09 - 4.70) pg/mL | 4.01 (3.90 - 4.32) pg/mL | p=0.913 | p=0.886 |
| IL-6 | Sedaghattalab 2021#2 | 58.24 (55.19 - 61.12) pg/mL | 56.57 (53.05 - 62.63) pg/mL | p=0.738 | 60.10 (55.99 - 73.10) pg/mL | 55.21 (53.39 - 60.48) pg/mL | **p=0.050** | p=0.196 |
| IL-1 | Shakerinasab 2023 | 1.06 (0.84 - 1.51) pg/mL | 0.84 (0.40 - 1.28) pg/mL | p=0.184 | 0.84 (0.40 - 1.28) pg/mL | 1.28 (0.62 - 1.95) pg/mL | **p=0.029** | **p=0.004** |
| CRP | Sedaghattalab 2021#2 | 10508.52 ± 5479.98 | 9104.60 ± 4901.67 | p=0.142 | 8953.3 ± 5588.06 | 7249.86 ± 5091.62 | **p=0.007** | p=0.785 |

**Appendix S3: Inflammatory markers reported across studies**

| Marker | Study | Placebo Pre-Treatment | Placebo Post-Treatment | Placebo Significance | Watercress Pre-treatment | Watercress Post-treatment | Watercress Significance | Watercress vs Placebo Significance |
| --- | --- | --- | --- | --- | --- | --- | --- | --- |
| Triglycerides | Clemente 2021 | NR | NR | NR | NR | NR | NR | No significance |
|  | Gill 2007 $ | 114 ± 71 mg/dL | 113 ± 64 mg/dL | NR | 113 ± 71 mg/dL | 100 ± 70 mg/dL | NR | NR |
|  | Sedaghattalab 2021 | 91.4 ± 59.6 mg/dL | 115.3 ± 96.3 mg/dL | **p<0.01** | 101 ± 57.1 mg/dL | 109.1 ± 51.8 mg/dL | p<0.24 | p<0.34 |
| Total Cholesterol | Clemente 2021 | NR | NR | NR | NR | NR | NR | No significance |
|  | Gill 2007 $ | 202 ± 39 mg/dL | 196 ± 36 mg/dL | NR | 198 ± 45.2 mg/dL | 189 ± 54.9 mg/dL | NR | NR |
|  | Sedaghattalab 2021 | 114 ± 27.4 mg/dL | 117.7 ± 33 mg/dL | p<0.42 | 130.2 ± 27.7 mg/dL | 129.3 ± 32.6 mg/dL | p<0.95 | p<0.62 |
| HDL | Clemente 2021 | NR | NR | NR | NR | NR | NR | No significance |
|  | Gill 2007 $ | 54.9 ± 17 mg/dL | 54.9 ± 15 mg/dL | NR | 56.8 ± 13.9 mg/dL | 54.9 ± 17 mg/dL | NR | NR |
|  | Sedaghattalab 2021 | 38 ± 12.3 mg/dL | 38.5 ± 9.9 mg/dL | p<0.64 | 37.7 ± 11 mg/dL | 38.9 ± 11.3 mg/dL | p<0.28 | p<0.31 |
| LDL | Clemente 2021 | NR | NR | NR | NR | NR | NR | **p<0.01** in post-intervention when compared to post-placebo |
|  | Gill 2007 $ | 121 ± 35 mg/dL | 117 ± 35 mg/dL | NR | 119 ± 37.5 mg/dL | 114 ± 41.8 mg/dL | NR | NR |
|  | Sedaghattalab 2021 | 51.6 ± 7.8 mg/dL | 58.2 ± 20.8 mg/dL | **p<0.04** | 65 ± 22.5 mg/dL | 64 ± 20.9 mg/dL | p<0.77 | p<0.2 |

**Appendix S4: Lipid markers reported across studies**
