# Supplementary material for: Evaluating the Anti‐Oxidant and Anti‐Inflammatory Properties of Watercress Supplementation at Short‐Term Follow‐Up: A Systematic Review of Randomized Controlled Trials
Source: Food Sci Nutr. 2025 Jun 5;13(6):e70407. doi: 10.1002/fsn3.70407 (PMC12141087; doi:10.1002/fsn3.70407)
Supplement: Supplementary file 2 — Table S1 [file FSN3-13-e70407-s002.docx]

| Author | Sequence Generation | Allocation Concealmeant | Blinding of Participants and Personnel | Blinding of Outcome Assessors | Incomplete Outcome Data | Selective Outcome Reporting | Other Source of Bias |
| --- | --- | --- | --- | --- | --- | --- | --- |
| Clemente 2020 | Low | Low | Low | Low | Low | Low | High |
| Clemente 2021 | Low | Low | Low | Low | Low | Low | High |
| Fogarty 2013 | Unsure | High | High | Unsure | Low | Low | Unsure |
| Gill 2007 | Unsure | High | High | High | Low | Low | High |
| Sedaghattalab 2021 | Unsure | Low | Unsure | Unsure | Low | Low | Unsure |
| Sedaghattalab 2021#2 | Unsure | Low | Unsure | Unsure | Low | Low | Unsure |
| Shakerinasab 2024 | Low | Low | Low | Low | Low | Low | Unsure |

Table S1: Cochrane Risk of Bias
